# Neural Mechanisms of Observational Learning: A Neural Working Model

**DOI:** 10.3389/fnhum.2020.609312

**Published:** 2021-04-22

**Authors:** Weixi Kang, Sònia Pineda Hernández, Jie Mei

**Affiliations:** ^1^Computational, Cognitive and Clinical Neuroimaging Laboratory, Division of Brain Sciences, Department of Medicine, Imperial College London, London, United Kingdom; ^2^Euncet Business School, Polytechnic University of Catalonia, Barcelona, Spain; ^3^Department of Anatomy, Université du Québec à Trois-Rivières, Québec City, QC, Canada

**Keywords:** cognitive flexibility, visuomotor learning, Imitation, vicarious learning, mirror system, observational learning, social learning, prefrontal cortex

## Abstract

Humans and some animal species are able to learn stimulus-response (S-R) associations by observing others' behavior. It saves energy and time and avoids the danger of trying the wrong actions. Observational learning (OL) depends on the capability of mapping the actions of others into our own behaviors, processing outcomes, and combining this knowledge to serve our goals. Observational learning plays a central role in the learning of social skills, cultural knowledge, and tool use. Thus, it is one of the fundamental processes in which infants learn about and from adults (Byrne and Russon, 1998). In this paper, we review current methodological approaches employed in observational learning research. We highlight the important role of the prefrontal cortex and cognitive flexibility to support this learning process, develop a new neural working model of observational learning, illustrate how imitation relates to observational learning, and provide directions for future research.

## Introduction

Observational learning refers to learning by watching others' actions and their associated outcomes in certain circumstances. Unlike imitation, observational learning does not simply mean to replicate an action that others have performed. Rather, it requires the learner to transform the observed scenarios into actions as close as possible to that of the actor's (Torriero et al., 2007). One of the most illustrative examples would be how Adelie penguins observe and learn from others' actions when congregating at the water's edge to enter the sea and feed on krill in Antarctica. However, the leopard seal—the main predator of the penguins—often hides beneath the waves, making it risky to be the first penguin to jump into the water. As the waiting game continues, one of the hungriest animals will jump into the water while others are watching. They will only follow if no seal appears (Burke et al., 2010). This ability to follow, to interpret and to learn from observed actions and outcomes is crucial for many species when the stakes are high. For example, predators can avoid eating poisonous prey without trying it by watching their peers consuming it (Burke et al., 2010). Many animal species, as well as human infants are born helpless and rely on observational learning (Meltzoff and Marshall, 2018). Research has found that newborns as young as 42 min are able to match gestures (including tongue protrusion and mouth opening) that has been shown to them (Meltzoff and Moore, 1997). Moreover, newborns can also map observed behaviors to their own, which suggests shared representation for the acts of self and others (Meltzoff and Moore, 1997; Meltzoff, 2007; Meltzoff and Marshall, 2018). Young infants can easily imitate, but only older infants demonstrate observational learning. In one study, a group of 14-month-old infants saw a novel act (e.g., the adult actor used his head to turn on a light panel). When encountering the same panel after a 1-week delay, 67% of infants used their head to turn on the light panel. This is observational learning because the infant reacts when seeing the stimulus by retrieving what they encoded in memory; rather than simply replicating an action (Meltzoff, 1988). Moreover, several studies from other groups have provided converging evidence for this, which found that infants not only copy goals and outcomes of the demonstration, but they also imitate the model used to attain that goal (e.g., Tennie et al., 2006; Williamson et al., 2008).

By definition, observational learning involves two main steps: (1) Infer other's intentions according to the observation, (2) process others' action outcomes (i.e., successes and errors) and combine these sources of information to learn the stimulus-response-outcome (S-R-O) associations that can be later used to obtain desirable outcomes. Unlike instruction-based learning (IBL; Hampshire et al., 2016), instruction is not required in observational learning, which would be advantageous for species which do not have language or aphasia patients who have difficulties comprehending linguistic instructions. Compared to widely investigated reinforcement learning (RL), observational learning enables one to acquire knowledge without taking the risks or incurring costs during discovering (Monfardini et al., 2008). This paper is structured in five main sections. First, we survey the current approaches in observational learning research. Next, we discuss the neural mechanisms underlying observational learning with a focus on the frontal-temporal system while comparing it to other forms of learning (e.g., IBL and RL). After that, we develop a neural working model of observational learning. We then connect observational learning with imitation and suggest that they should be regarded as two distinct cognitive processes. Finally, we discuss the role of ventral striatum in social learning and outline some possible future research directions.

## Schemes in Observational Learning Research

**Figure 1:**
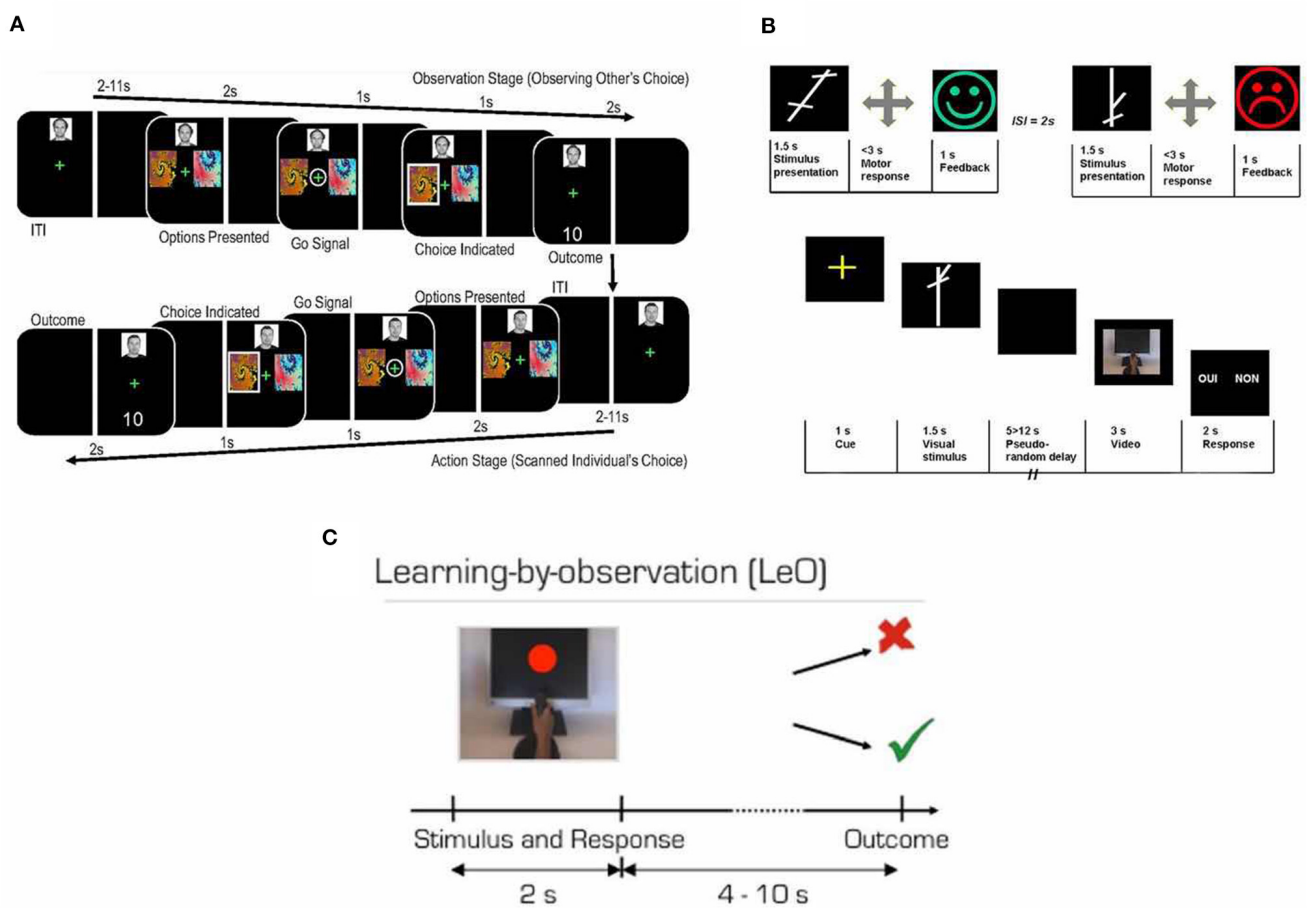
Experimental designs. **(A)** The upper panel, learning session. Participants learned the associations between abstract visual stimuli and corresponding joystick movements. In the experiment, the stimulus presented on the screen for 1.5 s, the participants were asked to move the joystick left, right, up, or down. Once participants made a response, a visual feedback was given to indicate if the response was correct (happy green face; example 1) or wrong (sad red face; example 2). The participants learned one set of S-R associations by trial-and-error exploration and the other by observing experts performing the task. The lower panel, scanning session. Trials in the three different conditions were pseudo-randomly intermixed. Across all conditions, the visual stimuli were presented for 1.5 s and followed by a blank screen of variable durations and a short video of an actor's hand performing a joystick movement. After the movie, participants were asked to judge whether the actor in the video made a correct response or not by pressing a right or left mouse corresponding to “yes” or “no” shown on the left or the right side of the screen, respectively. There was a 50% of change that the actor's response was correct with 50% of change that the actor's response was wrong. Adapted from Monfardini et al. (2008) with permission. **(B)** Prediction error approach paradigm. After a variable ITI, participants first were asked to observe the confederate players being presented with two abstract fractal stimuli to choose from. Then participants were presented with the same stimuli, and the trial proceeded with the same manner. Participants were asked to make a response when the fixation cross was circled by using the index finger for left stimulus and middle finger for right stimulus on the response pad. Adapted from Burke et al. (2010) under Creative Commons Attribution License (CC BY). **(C)** Each trial started with a video showing a hand on a joystick performing one of the four possible movements in response to the presentation of a colored stimulus on the monitor screen. The total video length was 2 s and the colored stimulus lasted 1.5 s. The outcome image was presented after a variable delay. Participants were instructed to learn the correct stimulus-response-outcome association by looking at the video and outcomes. Adapted from Monfardini et al. (2013) under Creative Commons Attribution License (CC BY).

There are three main kinds of task designs in current observational learning research (Figure 1). The first one was employed by Burke et al. (2010), who used the observational action prediction error (defined as the difference between actual choice and predicted choice of the other agent) and observational outcome prediction error (defined as the difference between the actual outcome and predicted outcome of the other agent's action, which were based on the widely used concept prediction error in reinforcement learning literature). More specifically, in a given trial of their experiment, participants had to choose between one of the two abstract fractal stimuli to gain a stochastic reward and to avoid stochastic punishment while being scanned by fMRI. One stimulus consistently delivered a good outcome (reward or absence of punishment 80% of the time) and bad outcome (punishment or absence of reward 20% of the time; Figure 1A). They also included a trial-and-error baseline individual learning condition and a learning from observing actions only condition which could be used to characterize the observational action prediction error. The second task design was introduced by Monfardini et al. (2008), in which participants watch a short video demonstrating an actor making motor responses according to the stimulus presentation with post-response feedback (Figure 1B). Importantly, in the fMRI scanning section, instead of participants themselves making their response when seeing the stimuli, participants watched the actor in the short video making a response according to the stimulus that participants saw before the video and made a binary choice regarding whether the actor made a correct response. This kind of design enables the detection of brain activity when participants retrieve rules (when novel stimuli are being displayed). However, logically, the part where participants had to watch an actor performing the task and make judgement about it is very unlikely to occur in a realistic situation because we seldomly learn from judging another, while judging others' responses to stimuli might involve additional cognitive processes. In a later study, Monfardini et al. (2013) changed the aforementioned design and introduced the learning by observation (LeO) task. In the LeO task, participants were asked to learn S-R associations between stimulus presented on the screen and joystick movements by watching a video which shows an expert demonstrating the correct visuomotor association. They were then asked to make responses accordingly in the fMRI scanner after each stimulus was presented (Figure 1C).

## Neural Mechanisms of Observational Learning

### Cognitive Flexibility and the Lateral Prefrontal Cortex

In uncertain and changing environments, flexible control of actions has evolutionary and developmental benefits as it enables goal-directed and adaptive behavior. Flexible control of actions require an understanding of the outcome (reward or punishment) associated with the given action (Burke et al., 2010), and it is well-established that cognitive flexibility plays an important role in both reinforcement learning and instruction-based learning. In reinforcement learning where trial-and-error exploration is commonly used, individuals can use the outcomes associated with previous actions to determine future actions (Thorndike, 1970; Mackintosh, 1983; Balleine and Dickinson, 1998; Skinner, 2019). In instruction-based learning, individuals can utilize learned rules and representations to choose the correct actions (Cole et al., 2013a). Theoretically speaking, cognitive flexibility is also required in observational learning as individuals learn the associations between other agents' actions and their associated outcomes and use this information to choose the correct responses rapidly.

The lateral prefrontal cortex (LPFC) is crucial when a high demand for cognitive flexibility is required to perform the task, e.g., during learning novel tasks (Cole et al., 2013a), decision making (McGuire and Botvinick, 2010), and task-switching (Braver et al., 2003; Ruge et al., 2013). Previous research has confirmed the essential role of LPFC in instruction-based learning in terms of transferring novel rules into execution rapidly (Cole et al., 2013a, 2016; Hampshire et al., 2016), where a high degree of cognitive flexibility was required. Studies on observational learning have also shown the involvements of the dorsolateral prefrontal cortex (DLPFC) and the ventrolateral prefrontal cortex (VLPFC; Monfardini et al., 2008, 2013; Burke et al., 2010) once the rule was learned, which is consistent with the notion that LPFC is engaged during acquisition of novel rules.

Note that the only difference between IBL and observational learning is that observational learning of novel rules requires subjects to learn by observing others' action and/or outcomes associated with that action whereas during IBL, subjects learn from explicitly linguistic or symbolistic instructions. According to previous research on IBL (Cole et al., 2013a), abstract IBL activates anterior LPFC while concrete IBL activates middle LPFC. In abstract IBL, participants were asked to judge if two words have the same property. For example, the stimulus might be “apple” and “grape,” and the participants were required to press down the left index finger if these two kinds of fruits were sweet and press down the right index finger if any of these two kinds of fruits was not sweet. In contrast, similar to the S-R associations in observational learning research, the concrete IBL tasks asked participants to press a button when seeing a novel stimulus. For instance, the instruction might ask the participants to press down the left index finger when seeing the shape “square” and to press down the right index finger when seeing the shape “circle.” Research has identified that the concrete IBL tasks activated more posterior areas of the LPFC and activation of LPFC shifted posteriorly with practice in both abstract and concrete IBL. One interpretation is that once the novel task becomes routine after a certain amount of practice, the task representation also becomes more concrete. Thus, we hypothesize that the more posterior parts of the LPFC will be activated during observational learning because watching someone performing the task would be more concrete than transferring linguistic rules into programmatic execution. Practice of novel tasks in observational learning will also lead to an anterior-posterior shift in LPFC. However, further studies are needed to confirm this hypothesis.

### Common Networks in Acquiring Rules

**Figure 2:**
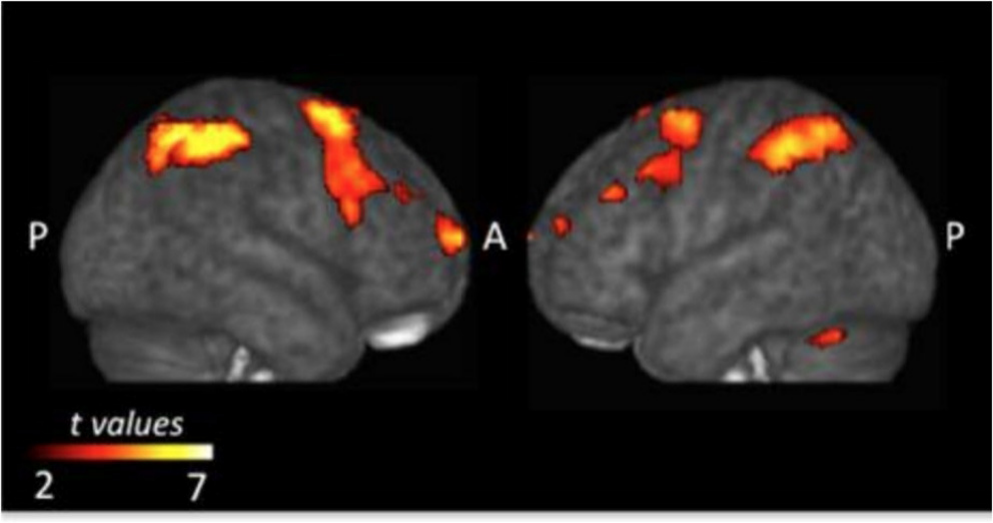
Common brain regions that were more active during the acquisition phase of both types of learning. Adopted from Monfardini et al. (2013) under Creative Commons Attribution License (CC BY).

Monfardini et al. (2013) found that it does not matter whether S-R associations are learned *via* observation or trial-and-error, three documented cerebral systems were involved in the acquisition stage: the dorsal frontoparietal, the fronto-striatal, and the cerebellar networks (Figure 2). The most straightforward interpretation is that during both types of learning, the brain builds a task model linking rule and response and it does not matter whether rules are acquired through observation or through trial-and-error. The dorsal frontoparietal system consists of the superior and inferior parietal lobes and the premotor dorsal cortex, which plays a central role in sensorimotor transformation, goal-directed attentional control to stimulus and response, and instrumental learning. Previous neuroimaging research has also confirmed its contribution to trial-and-error learning (Eliassen et al., 2003; Law et al., 2005) specifically during the processing of outcomes (Brovelli et al., 2008). This evidence suggested that processing of other's success and error during observational learning might involve the same brain systems as in individual learning, sensorimotor transformation, and goal-directed attentional control (Monfardini et al., 2013).

The frontostriatal networks comprise the left dorsal striatum, the anterior ventro-lateral, dorso-lateral prefrontal cortices and the supplementary motor area (SMA), which are considered to be of crucial importance for goal-directed operations during individual instrumental learning (Yin and Knowlton, 2006; Balleine et al., 2007; Graybiel, 2008; Yin et al., 2008; Packard, 2009; White, 2009; Ashby et al., 2010; Balleine and O'doherty, 2010). Previous research has observed activities in the caudate nucleus and the ventrolateral and dorsolateral frontal cortex during individual learning, and also in the premotor and supplementary motor areas (Frith and Frith, 1999, 2012; Toni and Passingham, 1999; Toni et al., 2001; Tricomi et al., 2004; Boettiger and D'Esposito, 2005; Delgado et al., 2005; Galvan et al., 2005; Seger and Cincotta, 2005; Grol et al., 2006; Haruno and Kawato, 2006; Brovelli et al., 2008). Specifically, the anterior caudate nucleus might integrate information about performance and cognitive control demand during individual instrumental learning (Brovelli et al., 2011), whereas the ventral lateral prefrontal cortex (VLPFC) is involved in the retrieval of visuomotor associations learned either by trial-and-error or by observation of others' actions (Monfardini et al., 2008). The fronto-striatal networks are also critical for adaptively implementing a wide variety of tasks where a high level of cognitive flexibility is required and these networks' ability to adapt to various contexts is made possible by the “flexible hubs,” which include neural systems that rapidly update their patterns of global functional connectivity depending on the task demands (Cole et al., 2013b).

The cerebellar network located bilaterally in the cerebellum was recruited in outcome processing at early stages of learning. Clinical evidence has shown that cerebellar lesions can give rise to impairments in procedural learning and cognitive planning (Grafman et al., 1992; Appollonio et al., 1993; Pascual-Leone et al., 1993; Gomez-Beldarrain et al., 1998). Moreover, a study that employed repetitive transcranial magnetic stimulation (rTMS; Torriero et al., 2007) has demonstrated the role of cerebellar regions in acquiring new motor patterns through both observation and trial-and-error. Monfardini et al. (2013) concluded that this network is involved in both observational learning and trial-and-error learning, even if there was no need to acquire new motor patterns.

### Common Networks in Retrieval of Associations

**Figure 3:**
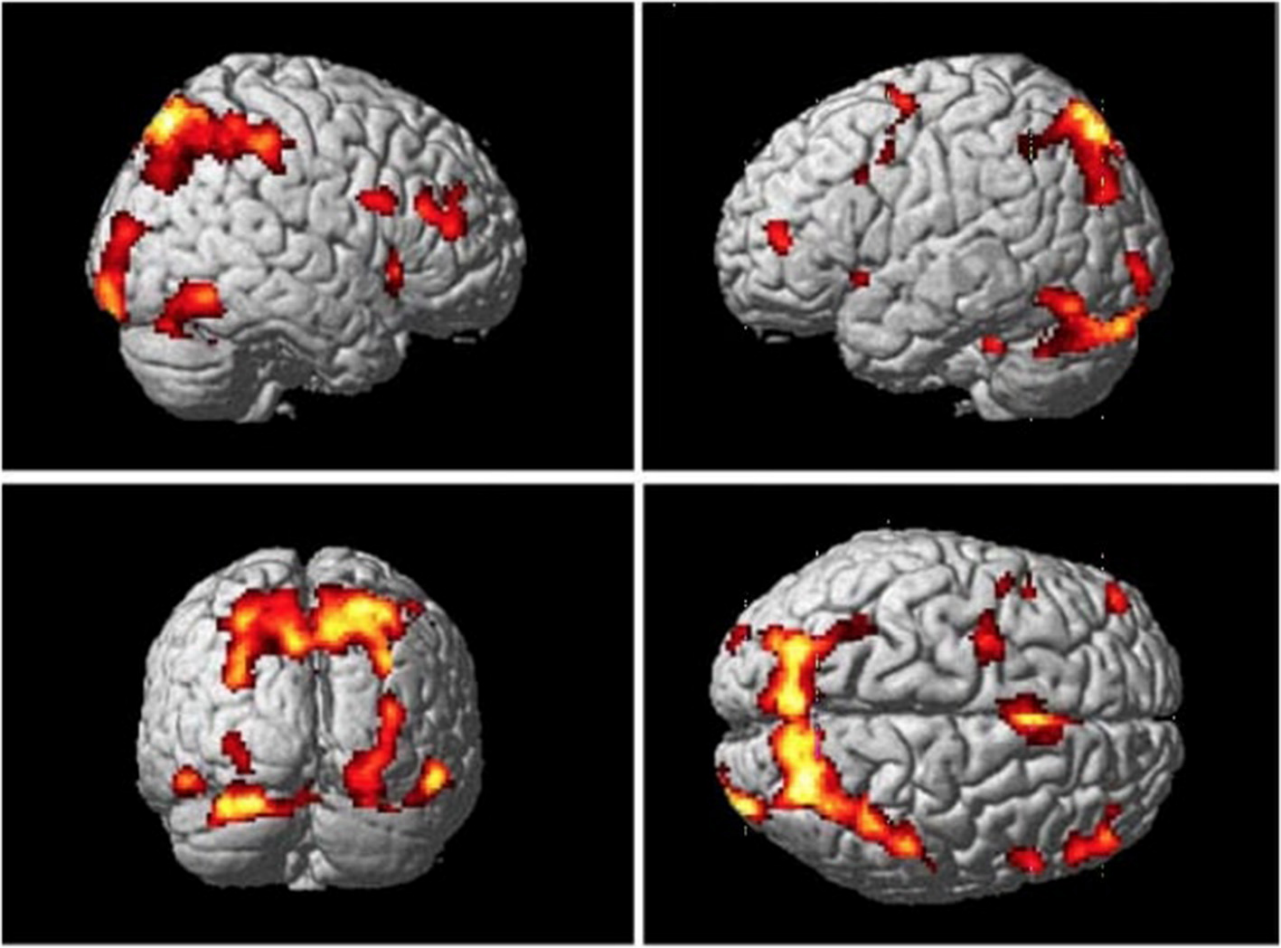
Common brain regions that were more active while participants viewing stimuli they had recently learned by either trial-and-error exploration or observation than control stimuli at the retrieval stage. Adopted from Monfardini et al. (2008) with permission.

S-R associations can be learned through instruction, observation, and trial-and-error, thus common networks involved in retrieval of associations are proposed. Results from the conjunction analysis in Monfardini et al. (2008) has demonstrated that when newly acquired rules were being retrieved, with the brain network consisting of the right ventrolateral and anterior frontal cortices, pre-SMA, as well as the parietal cortex, was highly involved (Figure 3; Bunge et al., 2003; Bunge, 2004; Donohue et al., 2005; Crone et al., 2006). This finding was consistent with the conclusions of Donohue et al. (2005) that assessed the contribution of the inferior frontal junction to the retrieval of motor responses associated with symbolic cues. It also suggested that the posterior medial temporal gyrus plays a crucial role in the representation of arbitrary associations (Donohue et al., 2005). The most straightforward interpretation of these findings is that during both observational learning and trial-and-error learning, the brain builds a task model which links rules with responses, thus involving brain networks that are commonly activated during retrieval of associations.

### Specific Networks for the Observational Learning

When retrieving rules learned through observation, the observation of abstract stimuli and their associations with corresponding responses activated a set of brain regions including the right pars triangularis (BA 45), the right inferior parietal lobule, and the posterior visual areas. Monfardini et al. (2008) compared the changes in BOLD signals in the right pars triangularis during retrieval with that during movie watching and identified similar patterns of activation when subjects retrieved motor responses associated with a visual arbitrary stimulus. This finding is consistent with the notion that the pars triangularis is engaged in observation of actions, but not in imitation or execution of actions (Molnar-Szakacs et al., 2005). Monfardini et al. (2008) proposed that the right pars triangularis should not be considered as part of the mirror system, rather, this brain region is related to the suppression of actions execution during both observation and motor imagery (Deiber et al., 1998; Molnar-Szakacs et al., 2006). Regarding activations in the posterior visual areas, Monfardini et al. (2008) suggested that it may be a result of top-down modulations. In line with this hypothesis, neuroimaging studies have proposed that during the execution of a given task, a frontal-parietal network exerts control over the activities in the visual cortex through top-down signals that modulate activities of the visual cortex. In Monfardini et al. (2008), attention to the hand was an instinct characteristic of observational learning. In the meantime, a retrieval process may reactivate the observed movements during the learning process and the top-down modulations may influence distinct visual areas, depending on whether the rules were learned through observation or trial-and-error processes (Super et al., 2001; Vidyasagar and Pigarev, 2007).

During implementation, many brain areas were significantly more active during the presentation of incorrect outcomes in observational compared to individual learning. In particular, the activated clusters of bilateral brain regions include the middle cingulate cortex (MCC), the posterior medial frontal cortex (pMFC), the anterior insula, and the posterior superior temporal sulcus (pSTS). Previous research has demonstrated that the pMFC and the anterior insula are both part of the error-monitor network (Radke et al., 2011). The pMFC is located within the dorsal anterior cingulate cortex, which has been found to play a central role in individual trial-and-error learning processes (Holroyd and Coles, 2002; Mars et al., 2005). A study by Monfardini et al. (2013) has also suggested that the pMFC is highly involved in both error-monitoring and subsequent behavioral adjustments. More specifically, it has been suggested that when adaptations are required according to the outcomes associated with an action, the performance-monitoring system in the pMFC implements as a signal of the need for adjustments (Ullsperger and Von Cramon, 2003). Recent results from electrophysiological experiments on monkeys suggested that neurons in the dorsomedial prefrontal cortex selectively respond to others' incorrect actions, and their patterns of activity are associated with the subsequent behavior adjustments (Yoshida et al., 2012). The anterior insular cortex is involved in both performance monitoring processes (Radke et al., 2011) and the autonomic responses to error in non-social contexts (Ullsperger and Von Cramon, 2003), and its level of activity increases with error awareness (Klein et al., 2007; Ullsperger et al., 2010). This network is also activated during error-detection and in non-learning contexts (Ridderinkhof et al., 2004; de Bruijn et al., 2009; Radke et al., 2011), however, there is no evidence suggesting its activities could differentiate others' from individual learning. Monfardini et al. (2013) provided novel insights into the role of the pMFC-anterior insular network in the processing of others' mistakes during observational learning. To date, a number of studies has established an association between the level of error-related activities and the subsequent learning performances (Klein et al., 2007; Hester et al., 2008; van der Helden et al., 2010). Monfardini et al. (2013) speculated that the neurons in the pMFC-anterior insular network may act as the neural correlates of a cognitive bias that have been referred to as the predisposition of humans to process errors of others differently from their own errors by studies in neuroeconomics and social psychology. Specifically, the “actor-observer” cognitive bias represents the tendency to attribute others' failures to their personal mistakes but one's own failure is attributed to the situation (Jones and Nisbett, 1987). For a deeper understanding of the relative effectiveness of individual and observational learning from others' and individual errors, further neuroimaging research is needed (Monfardini et al., 2013).

Monfardini et al. (2013) also demonstrated that the posterior superior temporal sulcus (pSTS) is specifically correlated with the processing of others' errors during observational learning. As previous studies on non-human primates showed that the STS is anatomically well-suited to integrating information sourced from both the ventral and dorsal visual pathways, in a number of studies, social cues in the STS region which is sensitive to stimuli that signal the actions of another individual were analyzed (Pandya and Yeterian, 1985; Boussaoud et al., 1990; Baizer et al., 1991). Particular emphasis has been given to the pSTS, which was regarded as the substrate of goal-directed behavior (Saxe et al., 2004) and social perception (Allison et al., 2000). Overall, previous research supports the hypothesis that perception of agency activates the pSTS (Tankersley et al., 2007), and the activity in pSTS might be part of a larger network mapping of observed actions to motor programs (Rilling et al., 2004; Keysers and Gazzola, 2006). Moreover, the pSTS is considered to be actively involved in the attribution of mental states to other organisms (Frith and Frith, 1999, 2003; Saxe and Kanwisher, 2003; Samson et al., 2004) and the extraction of contextual and intentional cues from goal-directed behavior (Toni et al., 2001). More importantly, activities of the pSTS have been observed in humans during imitation of actions (Iacoboni, 2005). Results from Monfardini et al. (2013) supported the hypothesis of the role of pSTS in the processing of social information, which is a necessary component of the learning stage of observational learning. Additionally, the fact that the pSTS was more activated by the errors made by others than one's own errors may imply more intensive mentalizing (e.g., what does the agent think now that they know that one certain action does not lead to positive outcomes?) or the reactivation of the visual representations of an observed action to decrease its association with the corresponding stimulus (Monfardini et al., 2013).

## Neural Working Model of Observational Learning

**Figure 4:**
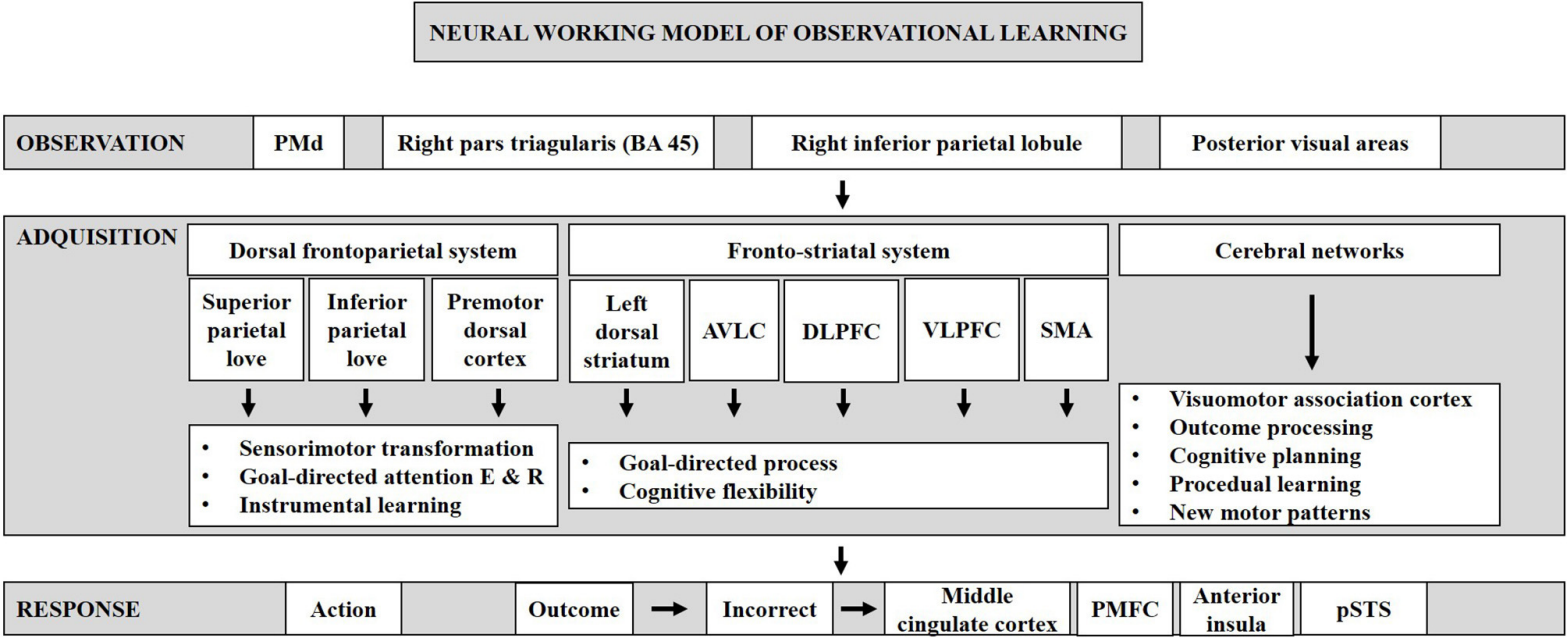
Neural working model of observational learning. It can be divided into three phases: observation, acquisition, and response.

To shed light on how observational learning works and its neural basis, we created a neural working model of observational learning (Figure 4). Our neural work model of observational learning includes three phases: observation, acquisition, response. The first phase is observation, defined as observation of abstract stimuli and their association to specific bodily movements, which activate a network consisting of a number of brain regions: the dorsal premotor cortex (Cisek and Kalaska, 2004), the right pars triangularis (BA 45), the right inferior parietal lobule, and the posterior visual areas (Monfardini et al., 2008). The ability to observe and learn with it is a powerful capacity of humans (Mattar and Gribble, 2005; Torriero et al., 2007), and previous studies have shown that when a symbolic representation of task performance is observed, neurons in the dorsal premotor cortex (PMd) respond in a similar manner than when the task is physically performed (Cisek and Kalaska, 2004).

The second phase is acquisition of rules, which recruits three individual systems: the dorsal frontoparietal, the fronto-striatal, and the cerebellar networks. The dorsal frontoparietal network consists of the bilateral superior and inferior parietal lobes and the premotor dorsal cortices, which are thought to involve in sensorimotor transformation, in the regulation of goal-directed attention to both stimulus and response, and in instrumental learning (Monfardini et al., 2013). The fronto-striatal network consists of the left dorsal striatum, the anterior ventro-lateral, and dorso-lateral prefrontal cortices, ventral lateral prefrontal cortex, and the SMA. During instrumental learning, this network is thought to facilitate goal-directed processes (Yin and Knowlton, 2006; Balleine et al., 2007; Graybiel, 2008; Yin et al., 2008; Packard, 2009; White, 2009; Ashby et al., 2010; Balleine and O'doherty, 2010). In early phases of learning, the cerebellar network, located bilaterally in the cerebellum, is involved in outcome processing and assessment. It is also related to cognitive planning and procedural learning. Moreover, the cerebellar structures play a role in the acquisition of new motor patterns learned through observation (Grafman et al., 1992; Appollonio et al., 1993; Pascual-Leone et al., 1993; Gomez-Beldarrain et al., 1998).

The third phase is the response where the learner retrieves learned S-R contingencies and makes a response when seeing the stimulus. After making a response, if the outcome is incorrect, then it activates the error-monitoring network comprised of the middle cingulate cortex, the posterior medial frontal cortex (pMFC), the anterior insula, and the posterior superior temporal sulcus (pSTS; Radke et al., 2011). Usually, action and outcome are accompanied by a shift from feedback-based performance to response-based performance, and action and outcome can be learned well, both actively and by observation (Bellebaum and Colosio, 2014).

## Observational Learning vs. Imitation

Previous studies on observational learning have focused on the learning of novel motor patterns through imitation and mirror-like mechanisms (Monfardini et al., 2013). In imitation tasks used in these studies, participants did not need to search for the correct response to the current stimuli from multiple observations. Instead, they were required to imitate other people's actions regardless of the outcomes. Classical imitation research has suggested that the main circuitry of imitation consists of the superior temporal sulcus and the mirror neuron system, which includes the posterior inferior frontal gyrus, and ventral premotor cortex, and the rostral inferior parietal lobule [see Iacoboni (2005) for a review]. Several studies have found that the frontoparietal putative mirror neuron system (pMNS), which consists of the ventral and dorsal premotor cortex, the inferior parietal lobule and adjacent somatosensory areas, and the middle temporal gyri, was strongly activated while participants were observing others' actions during the acquisition of motor patterns (Caspers et al., 2010). Moreover, the pMNS was also recruited when participants were watching others' actions without the need to imitate, or they simply executed those actions (Monfardini et al., 2013).

However, the role of pMNS is ambiguous in observational learning of arbitrary visuomotor associations because the differentiation between actions that would result in positive feedback and actions that lead to negative feedback remain unexplored. In observational learning tasks, no novel motor responses have to be acquired during learning stages. Instead, new associations have to be established between the stimulus presented, acquired motor responses, and the associated outcomes. Monfardini et al. (2013) found that in line with activities of the pMNS, as outlined in the literature, both observational learning and individual trial-and-error learning activated a brain network that was also involved in simple action execution and observation. The increase in activations following outcome presentation was larger in observation learning than in trial-and-error learning in most trials. However, during the LeO task, the BOLD signal was larger in practice than in observation. The comparatively lower activation during the observation stage compared to the practice stage is a common finding in studies on pMNS and might be explained by the fact that only ~10% of premotor neurons respond to action observations in primates (Gallese et al., 1996; Keysers et al., 2003). It is therefore challenging to infer why observational learning induces a slightly larger BOLD signal than trial-and-error learning does in somatosensory motor regions. Researchers hypothesized that the BOLD signal in the somatosensorimotor regions were more activated in LeO due to the fact that unlike in the trial-and-error learning condition, responses were not given by the participants during the S-R acquisition stage (Monfardini et al., 2013). Thus, participants might be strong to mentally re-enact the observed response upon knowing whether it was to be associated with the stimulus or not. Without overt execution, it would be important to use additional mental re-enactments of others' actions to consolidate the S-R association that needs to be established during the learning process. This notion is consistent with proactive control in instruction-based learning, where goal-relevant information is actively maintained in preparation for the anticipated high control demand (Cole et al., 2018).

## Ventral Striatum and Social Learning

Burke et al. (2010) found that the ventral striatum, a brain region that has been found to be frequently related with the processing of prediction errors in individual instrumental learning, showed the inverted coding patterns for observational prediction errors. Despite the fact that Burke et al. (2010) did not present participants with a game situation and there was no way that the behavior of the confederate would affect the probability of participants obtaining the reward, this inverse reward prediction error encoding of the confederate's behavioral outcomes was supported by previous studies emphasizing the critical role of the ventral striatum in competitive situations. Nevertheless, the following explanations must be carefully assessed, given that Burke et al. (2010) did not include non-social control trials in their task design. For example, the ventral striatum is involved when a competitor is punished (e.g., received less money than oneself). This gives rise to several future research directions including the role of the ventral striatum in learning from each other. In the meantime, are positive reward prediction errors a sophisticated result of viewing others' loss during observational learning, or it is simply rewarding to see the misfortune of others? Recent data suggested the perceived similarity between the personalities of the participant and the confederate modulates activities in the ventral striatum when observing a confederate succeed in a non-learning task. In action-only learning scenarios, the individual outcome prediction error signals from the ventral striatum not only increase the selection accuracies of one's own outcome-oriented choices but can also refine predictions of others' choices based on information about their past actions (Burke et al., 2010).

## Concluding Remarks

In conclusion, observational learning is an important cognitive process in both animals who do not have language and humans whose infants imitate and learn from adults during development and early learning stages. We surveyed three kinds of methodological approaches in the investigation of this cognitive process, with emphasis on different stages of learning as well as on different modeling aspects. LPFC is essential for cognitive flexibility, which is required in observational learning where subjects have to rapidly learn rules from observing others' actions and outcomes associated with these actions. Trial-and-error learning and observational learning share some networks both during acquiring rules and applying rules, although observational learning also involves some additional networks. A neural working model has been developed for observational learning consisting of three phases: observation, acquisition, and response. This model is important because it disentangled the sub-processes and neural systems involved in observational learning. It provides foundations for future cognitive neuroscience and translational clinical research. Observational learning is different from imitation, both conceptually (observational learning involves processing of others' actions and outcomes to know how to react when encountering the same situation, whereas imitation only requires the subject to replicate others' actions) and neuroscientifically (the mirror neuron system is thought to be critical in imitation and is only partly activated in observational learning).

Future research questions include (a) how the observational learning processes in sports could be optimized to facilitate motor skill acquisition and improve performance, (b) how the brain activity patterns shift with practice in observational learning, and (c) how observational learning is employed in human infants that have not fully developed language abilities and how the brain supports this cognitive process. Imitation has been studied in children with the aim to understand its relationship with development (Sebastianutto et al., 2017). However, relatively little attention has been paid to observational learning from a developmental perspective. Unlike adults, neuroimaging methods such as fMRI and transcranial magnetic stimulation (TMS) cannot be applied to infants, making it challenging to directly analyze brain activation patterns in the study of observational learning. Previous works using electroencephalogram (EEG) have elucidated the properties of the mu rhythm in infants during imitation (e.g., Marshall and Meltzoff, 2014), and it would be important for future studies to investigate how observational learning relates to development in infants and how observational learning differs from imitation in infants using EEG.

As observational learning and imitation allow for scenario-specific adaptive behavior, learning and task execution, increasing interest has been shown in the application of findings in both fields to computational modeling, artificial intelligence, and robotics (Liu et al., 2018). More specifically, research has demonstrated that biologically inspired learning models could be implemented to enable robotic learning by imitation and allow for human-robot collaboration (Chung et al., 2015). Furthermore, modeling of imitation and observational learning using robots facilitates testing and refinement of hypotheses in developmental psychology and human-robot interaction. In one previous study, developmental robots were used to model human cognitive development and to examine how learning could be achieved through interaction that involves mutual imitation (Boucenna et al., 2016). Taken together, in future studies, it would be of considerable relevance to examine how understanding of observational learning processes and imitation could lead to advancements in the fields of robotics and cognitive modeling.

From a clinical-translational perspective, previous research has revealed that functional connectivity of some of the LPFC networks are impacted in patients who suffer from traumatic brain injuries (Hampshire et al., 2013) or patients with neurodegenerative disorders (Grafman et al., 1992). As an example, patients with Parkinson's disease who had abnormal striatum function showed slower acquisition of contingency reversal learning paradigms (Williams-Gray et al., 2008). Therefore, another sensible future direction is to determine whether the connectivity effects observed during simple observational learning can provide clinical diagnostic markers in populations that suffer from cognitive impairments.

### Limitations

The current review focused on neuroimaging literature of observational learning using healthy participants. Other methodologies (e.g., electrophysiology and lesion studies) may further confirm the role of each brain region in observational learning in our review. Moreover, a review on studies from atypical participants (e.g., infants, aging adults, people with neurological conditions) may provide further insights on the relevance of observational learning to other aspects (e.g., developmental theories and rehabilitation).

## Author Contributions

WK: conceptualization, writing–original draft, visualization, writing–review & editing, and funding acquisition. SP: conceptualization, writing–original draft, and visualization. JM: writing–review & editing. All authors contributed to the article and approved the submitted version.

## Conflict of Interest

The authors declare that the research was conducted in the absence of any commercial or financial relationships that could be construed as a potential conflict of interest.
